# Detection of Dental Anomalies in Digital Panoramic Images Using YOLO: A Next Generation Approach Based on Single Stage Detection Models

**DOI:** 10.3390/diagnostics15151961

**Published:** 2025-08-05

**Authors:** Uğur Şevik, Onur Mutlu

**Affiliations:** 1 Department of Computer Science, Faculty of Science, Karadeniz Technical University, Kanuni Campus, 61080 Trabzon, Turkey; onurmutlu@ktu.edu.tr; 2 Retina R&D Software and Engineering Services Ltd., Trabzon Teknokent, 61080 Trabzon, Turkey

**Keywords:** pediatric dentistry, YOLO models, panoramic radiographs, automated detection, deep learning, clinical decision support

## Abstract

**Background/Objectives**: The diagnosis of pediatric dental conditions from panoramic radiographs is uniquely challenging due to the dynamic nature of the mixed dentition phase, which can lead to subjective and inconsistent interpretations. This study aims to develop and rigorously validate an advanced deep learning model to enhance diagnostic accuracy and efficiency in pediatric dentistry, providing an objective tool to support clinical decision-making. **Methods**: An initial comparative study of four state-of-the-art YOLO variants (YOLOv8, v9, v10, and v11) was conducted to identify the optimal architecture for detecting four common findings: Dental Caries, Deciduous Tooth, Root Canal Treatment, and Pulpotomy. A stringent two-tiered validation strategy was employed: a primary public dataset (*n* = 644 images) was used for training and model selection, while a completely independent external dataset (*n* = 150 images) was used for final testing. All annotations were validated by a dual-expert team comprising a board-certified pediatric dentist and an experienced oral and maxillofacial radiologist. **Results**: Based on its leading performance on the internal validation set, YOLOv11x was selected as the optimal model, achieving a mean Average Precision (mAP50) of 0.91. When evaluated on the independent external test set, the model demonstrated robust generalization, achieving an overall F1-Score of 0.81 and a mAP50 of 0.82. It yielded clinically valuable recall rates for therapeutic interventions (Root Canal Treatment: 88%; Pulpotomy: 86%) and other conditions (Deciduous Tooth: 84%; Dental Caries: 79%). **Conclusions**: Validated through a rigorous dual-dataset and dual-expert process, the YOLOv11x model demonstrates its potential as an accurate and reliable tool for automated detection in pediatric panoramic radiographs. This work suggests that such AI-driven systems can serve as valuable assistive tools for clinicians by supporting diagnostic workflows and contributing to the consistent detection of common dental findings in pediatric patients.

## 1. Introduction

Oral diseases represent a major global public health issue, affecting individuals across their lifespan and diminishing their overall quality of life through pain, functional limitations, and psychosocial distress [1]. According to the WHO’s 2022 Global Oral Health Status Report, these conditions affect nearly half of the world’s population, with an estimated 3.5 billion people impacted, showing a higher prevalence in low and middle income countries [2]. The economic consequences are profound, encompassing not only high direct treatment costs but also significant indirect costs related to lost productivity from work and school [3]. Among the spectrum of oral diseases, untreated dental caries of permanent teeth is the single most prevalent condition, affecting over 2 billion people globally, underscoring its status as a primary target for diagnostic and preventive strategies.

Children are a particularly vulnerable demographic for oral diseases due to factors such as cariogenic dietary habits, developing oral hygiene practices, and the unique anatomical features of primary teeth. The impact is significant; for instance, a study in the United States revealed that 43.1% of children aged 2–19 are affected by dental caries [4]. The consequences of untreated caries in this age group extend beyond the need for invasive procedures like Root Canal Treatment or tooth extraction. They can also lead to malnutrition, speech impediments, poor school performance, and diminished self-esteem. Therefore, early and accurate diagnosis is paramount, yet conventional visual tactile examinations can be challenging, especially for detecting incipient lesions on complex surfaces. This diagnostic gap highlights the urgent need for advanced technological aids.

The cornerstone of traditional dental diagnosis is a combination of clinical examination and radiological imaging. Among various radiographic techniques, panoramic radiography (OPG) is a fundamental tool, particularly in pediatric dentistry, as it provides a comprehensive overview of all teeth, the mandible, and the maxilla in a single image [5,6]. This broad perspective is invaluable for assessing dental development, identifying gross pathological lesions, and formulating holistic treatment plans [7]. However, OPGs are not without limitations; they are susceptible to image distortion, overlapping of anatomical structures, and have a lower spatial resolution compared to intraoral radiographs, which can make the detection of subtle anomalies challenging for the human eye [5].

The diagnostic utility of these images is heavily reliant on human interpretation, a cognitive task that requires significant expertise and is susceptible to factors like diagnostic fatigue and high inter and intra observer variability. This subjectivity can lead to inconsistent diagnostic outcomes. To address these challenges, artificial intelligence has emerged as a transformative technology in medical imaging, offering the potential to augment human capabilities by providing objective, rapid, and consistent analysis of complex radiological data.

Within the broader field of artificial intelligence, deep learning, and specifically Convolutional Neural Networks (CNNs), have become the predominant methodology for medical image analysis [8,9,10]. Unlike traditional machine learning approaches that require manual feature engineering [9], CNNs are designed to automatically learn hierarchical feature representations directly from raw image data [11,12,13]. The application of these technologies, which provide powerful support to clinicians [14,15], is especially critical in pediatric dentistry. This field is characterized by the dynamic landscape of the mixed dentition phase, where the constant state of change in pediatric radiographs presents a unique challenge. Developing tooth germs and resorbing roots can be mistaken for pathology, highlighting the urgent need for intelligent systems trained to differentiate these complex variations from true anomalies [16,17].

While studies have successfully applied AI for singular tasks in dentistry, such as detecting permanent tooth germs [18] or automating deciduous tooth numbering [19], a review of the literature reveals two critical gaps. Firstly, most existing studies focus on either adult populations or single, well-defined tasks, leaving the challenge of simultaneous, multi-class detection in the complex pediatric environment underexplored. Secondly, the practical clinical efficiency and inference speed of the proposed models are often secondary considerations. To address these gaps, this study aims to develop and validate a model for the simultaneous detection of multiple common pediatric findings. Furthermore, recognizing the need for a balance between speed and accuracy in a clinical setting, we conduct a rigorous comparative evaluation of several state-of-the-art, single-stage object detection algorithms from the YOLO family to identify the most effective architecture for this multifaceted task.

## 2. Materials and Methods

### 2.1. Dataset and Preprocessing

**Figure 1:**
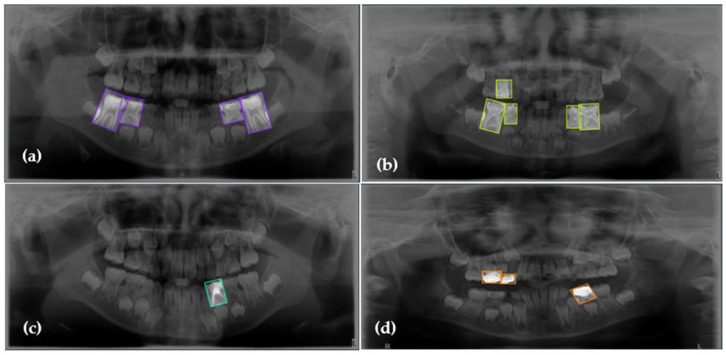
Representative examples of the four annotated dental conditions from the primary dataset. The images display examples of (**a**) Dental Caries (purple boxes), (**b**) Deciduous Tooth (green boxes), (**c**) Root Canal Treatment (cyan box), and (**d**) Pulpotomy (orange boxes). Bounding boxes indicate the ground-truth labels used for model training, with each color corresponding to the specific condition shown in the respective panel.

This study’s methodology is built upon a rigorous two-tiered data strategy using two distinct image cohorts. The primary dataset, used for model training and internal validation, was sourced from the publicly available collection of pediatric panoramic images by Zhang et al. From an original set of 644 images, a focused dataset was curated comprising the four common and radiographically distinct findings relevant to this study, with a detailed class distribution presented in Table 1 and visually represented in Figure 1. For the final, unbiased assessment of the model’s generalization capabilities, a separate external test dataset was curated, consisting of 150 pediatric panoramic images sourced from various public projects on the Roboflow Universe platform further details on the implementation of this external validation set are provided in Section 2.3.1. This dual-cohort approach ensures a comprehensive and robust evaluation pipeline.

#### Image Quality Assessment and Selection Criteria

To ensure the high fidelity of both the training and testing datasets, every OPG underwent a rigorous, multi-stage quality assessment conducted by the two clinical experts. This systematic protocol was designed to standardize the selection process and eliminate the subjectivity of general quality descriptors. An image was included in the final dataset only if it met all of the following inclusion criteria:

- Complete Anatomical Coverage: The radiograph had to provide a complete view of the dentition, extending from the right third molar region to the left third molar region, while clearly visualizing both mandibular condyles and the inferior border of the mandible.
- Correct Patient Positioning: Images had to exhibit proper patient positioning, characterized by a slight “smile line” of the occlusal plane and no superimposition of the spinal column over the anterior teeth.
- Sufficient Image Contrast and Density: The image density and contrast levels had to be adequate to allow for clear differentiation between enamel, dentin, and pulpal tissues.
- Conversely, images were excluded if they presented one or more of the following significant issues:
- Major Positioning Errors: Severe errors such as a slumped patient position or incorrect head tilt that resulted in significant distortion or magnification of the dental arches.
- Obscuring Artifacts: Presence of artifacts from earrings, eyeglasses, or lead aprons that superimposed over critical anatomical structures and impeded diagnostic evaluation.
- Motion Artifacts: Blurring across the majority of the image due to patient movement during exposure, rendering fine details diagnostically unusable.

Following the strict application of these objective criteria, all 644 primary and 150 external images were confirmed to be of diagnostically acceptable quality, forming a reliable foundation for model development and validation.

Following the dataset curation, a two-stage preprocessing pipeline was applied to prepare the images for model training. First, the annotation process was carried out using Label Studio, an open-source data labeling tool. Bounding boxes were drawn around each of the four target conditions by the initial annotator. Second, to meet the input requirements of the YOLO architecture and ensure computational efficiency, all images were resized to a uniform dimension of 640 × 640 pixels. Finally, the pixel values of all images were normalized to a range of [0, 1] to standardize the data and accelerate model convergence during training.

It is important to clarify the definition of the ‘Dental Caries’ category used in this study. The label was established to encompass both active carious lesions, which appear as radiolucencies, and completed dental restorations, which are typically radiopaque. This unified approach was adopted because both findings are clinically significant sequelae of the caries disease process and the original public dataset did not consistently differentiate between these two states. Therefore, for clarity and consistency throughout the remainder of this manuscript, the term ‘Dental Caries’ will be used to refer to this combined category.

Furthermore, it is critical to define the specific radiographic criteria used for the “Root Canal Treatment” and “Pulpotomy” categories. We acknowledge that diagnoses from panoramic radiographs are inferred from 2D radiodense patterns, not from direct clinical confirmation. Therefore, for the purposes of this study, our labels were based on distinct radiographic appearances. The ‘Root Canal Treatment’ label was applied to teeth exhibiting radiopaque material filling the intracanal space, a pattern consistent with endodontic therapy. This radiographic definition may pragmatically include similar presentations, such as teeth that have undergone apexification. Similarly, the ‘Pulpotomy’ label was assigned exclusively to primary teeth showing a distinct radiopaque base material within the coronal pulp chamber. This pattern-based labeling strategy was intentionally chosen to align with the operational logic of a computer vision model, which learns to recognize visual features, ensuring our ground truth was both consistent and defensible.

To establish an ideal ground truth for all data used in this study, both the primary public dataset (*n* = 644 images) and the independent external dataset (*n* = 150 images) were independently evaluated by two experienced clinicians: an oral and maxillofacial radiologist with over 10 years of experience and a board-certified pediatric dentist. To manage any potential discrepancies and address the lack of a tie-breaker with an even number of annotators, the study protocol included a predefined conflict resolution strategy: any annotation where the two experts disagreed on the label’s class or bounding box coordinates would be adjudicated by a third, senior reviewer (a professor in oral and maxillofacial radiology) to reach a final consensus. However, throughout the evaluation process, no disagreements arose between the two primary experts. The subsequent formal inter-rater reliability analysis confirmed this, yielding a Cohen’s Kappa coefficient of κ = 1.00, which represents perfect agreement. This outcome obviated the need for third-reviewer intervention and provided absolute confidence in the reliability of all annotations used for training and testing.

### 2.2. Model Selection and Architecture

Modern object detection has evolved significantly from early methods based on hand-designed features, such as Viola-Jones [21] and the Histogram of Oriented Gradients [22]. The advent of Convolutional Neural Networks (CNNs), marked by the success of AlexNet [23] revolutionized the field, paving the way for applications ranging from autonomous vehicles to medical image analysis. Current deep learning models are broadly categorized into two-stage detectors like Faster R-CNN [24], which are noted for high accuracy, and single-stage detectors like You Only Look Once (YOLO) [25], favored for their real-time processing speed. Furthermore, the field has produced architectures optimized for computational efficiency, such as EfficientDet [26], and foundational models like the Swin Transformer [27], which introduced new paradigms for image analysis beyond traditional CNNs. Given the need for a balance between rapid inference and high accuracy in a clinical setting, this study selected the YOLO framework, a state of the art single-stage detector.

The selection of the YOLO framework was a deliberate methodological choice made after considering other prominent computer vision paradigms. Our approach prioritized object detection over semantic segmentation techniques (e.g., U-Net, Mask R-CNN) for two primary reasons. First, the clinical objective was the rapid detection and localization of dental findings, a task for which bounding boxes provide sufficient and actionable information for initial diagnosis. The pixel-level precision offered by segmentation, while valuable for other applications like volumetric analysis, requires a significantly higher annotation burden, making large-scale dataset creation less feasible. Second, within the object detection domain itself, the single-stage YOLO architecture was chosen over two-stage detectors (e.g., Faster R-CNN). While two-stage models are renowned for high accuracy, single-stage detectors like YOLO offer a superior balance of speed and performance, a critical advantage for integration into time-sensitive clinical workflows. Finally, modern YOLO architectures are well-equipped to handle complex scenes, such as mixed dentition, through advanced feature fusion networks in their ‘Neck’ component, which effectively process objects at multiple scales and help to resolve issues of overlap.

**Figure 2:**
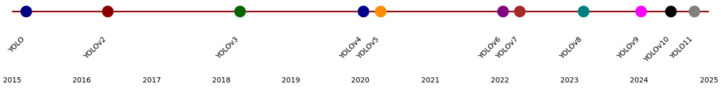
Timeline of the major YOLO architecture releases from 2015 to 2024 [28].

The YOLO architecture, first introduced in 2015, has continuously evolved to optimize the trade-off between detection speed and accuracy. As illustrated in its developmental timeline (Figure 2), later versions have incorporated more complex and efficient components to enhance real-time performance and the detection of small objects. To ensure the use of a state of the art model for this study, we evaluated the most recent iterations of the YOLO family: YOLOv8, YOLOv9, YOLOv10, and YOLOv11. To select the most suitable variant for our task, we compared the high-performance versions of these models (YOLOv8x, YOLOv9e, YOLOv10x, and YOLOv11x) based on their parameter counts and computational cost. The computational cost was measured in FLOPs (Floating-point Operations per Second), a metric that quantifies the number of calculations required for a prediction; a lower FLOPs value generally indicates a more computationally efficient and faster model. These comparisons are detailed in Table 2. This comparison highlighted the need for empirical evaluation, as architectural efficiency (e.g., YOLOv10x having fewer parameters than YOLOv11x) does not always directly translate to superior performance on a specific task.

The evaluated models in the YOLO series (v8, v9, v10 and v11) share a foundational three-part structure comprising a Backbone, Neck, and Head, but they introduce distinct architectural innovations aimed at improving the speed accuracy trade off. YOLOv8 features a streamlined and efficient architecture, making it a strong baseline. YOLOv9 introduced significant enhancements like Programmable Gradient Information and the Generalized Efficient Layer Aggregation Network to improve information flow and learning efficiency. Subsequently, YOLOv10 redesigned the model by incorporating dual label assignments and, most notably, achieving an end to end, NMS free pipeline for deployment, which reduces inference latency. The YOLOv11 model, which is a primary focus of this study, builds upon these advancements by integrating transformer based attention modules and more advanced feature fusion strategies in its neck component to further enhance the detection of small and varied objects. This study’s empirical comparison aims to determine how these progressive architectural modifications affect diagnostic performance on pediatric OPG’s.

### 2.3. Model Training and Parameters

#### 2.3.1. Model Training and Validation Strategy

**Figure 3:**
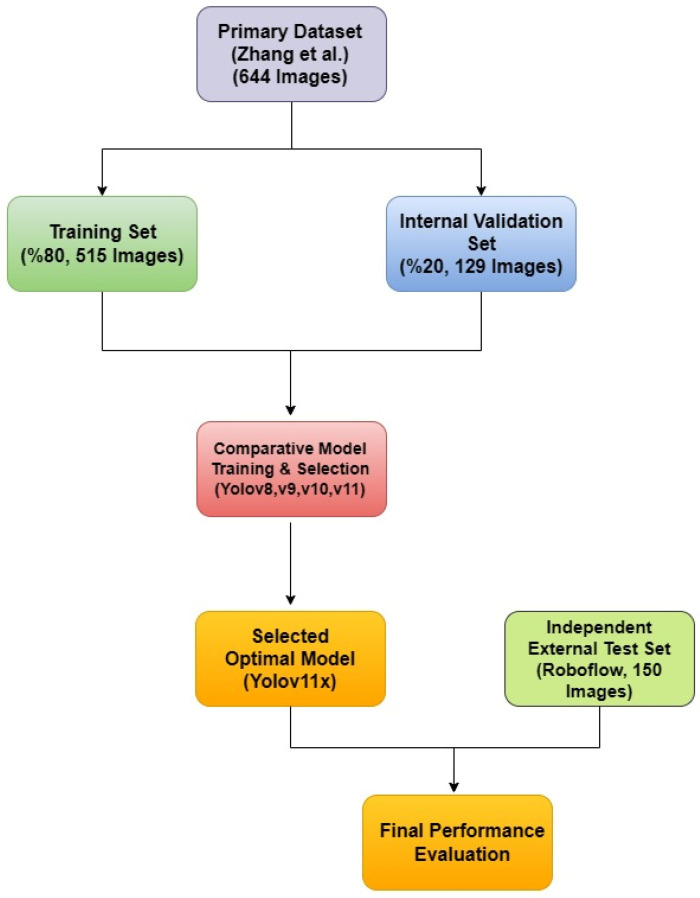
The diagram illustrates the two-tiered validation strategy, showing the use of a primary dataset for model training and selection, and a separate, independent external dataset for the final performance evaluation of the selected mode [20].

A rigorous, two-tiered validation approach was designed to first optimize the model parameters using the primary dataset and then to provide an unbiased assessment of its generalization capabilities on a completely independent, external dataset. This strategy directly addresses the limitations of a simple fixed-split methodology and provides a robust evaluation of the model’s real-world performance. This entire methodological workflow is visually summarized in Figure 3.

- Tier 1: Internal Training and Hyperparameter Tuning on the Primary Dataset

The public dataset of 644 images provided by Zhang et al. was utilized exclusively for the training and internal validation phases. Given that OPG’s in this dataset can contain multiple different labels (a multi-label problem), an iterative stratification splitting strategy based on labels was adopted to ensure a representative distribution. The dataset was partitioned into a training set (80%, 515 images) and a validation set (20%, 129 images), with this method guaranteeing that the proportions of each of the four annotated classes were preserved across both subsets. The training set was used for learning the model’s weights, while the validation set was essential for monitoring overfitting, tuning hyperparameters, and selecting the best-performing model checkpoint during the training process.

- Tier 2: External Validation on an Independent Public Dataset

To conduct a true and unbiased test of the finalized model’s ability to generalize to data from a different source, a new, independent test cohort of 150 pediatric OPG’swas meticulously curated. This external validation set was sourced from several large, publicly available projects on the Roboflow Universe platform. The specific data sources used for curating this test set are explicitly listed in the ‘Data Availability’ section to ensure full transparency and facilitate reproducibility. Great care was taken to select images that matched our study’s inclusion criteria and to ensure that there was absolutely no overlap between this external test set and the primary dataset used for training and validation.

To maintain a consistent and high-quality evaluation standard, this new cohort of 150 images was subjected to the same preprocessing pipeline and rigorous annotation protocol as the primary dataset. The images were independently annotated and verified by our two experts to ensure the ground truth was consistent with our study’s diagnostic criteria. This external test set was strictly held out and used only once for the final performance evaluation. Testing the model on data originating from different clinics, imaging equipment, and patient populations, as provided by this external cohort, offers a powerful and realistic measure of the model’s true generalization performance and clinical potential.

#### 2.3.2. Training Configuration and Hyperparameters

To ensure a fair and standardized comparison, all evaluated YOLO models were trained from scratch using a consistent set of hyperparameters, detailed in Table 3. These parameters were selected based on a combination of the Ultralytics framework’s established baseline configurations and best practices reported in the object detection literature. To enhance model generalization and prevent overfitting, a standard suite of data augmentation techniques including mosaic, mixup, and random affine transformations was applied to the training data. The models were trained for 500 epochs to ensure convergence, with performance on the validation set monitored throughout the process to select the optimal checkpoint for each model.

### 2.4. Performance Evaluation Metrics

The performance of the trained models was quantitatively evaluated using a comprehensive set of metrics. These metrics were chosen to assess the model’s optimization during training (loss functions), its core classification capability (classification metrics), and its overall effectiveness in object detection (detection metrics).

#### 2.4.1. Loss Functions

The model’s learning process was guided by minimizing a composite loss function, which is a weighted sum of three key components:

- Bounding Box (Box) Regression Loss: This component quantifies the accuracy of the predicted bounding box locations and sizes. Modern YOLO versions utilize advanced IoU-based losses, such as Complete IoU (CIoU) Loss, which accounts for overlap area, central point distance, and aspect ratio, leading to more stable training.
- Classification (CLS) Loss: This measures the correctness of the class predictions for each detected object. It is typically calculated using Binary Cross-Entropy (BCE) with logits, which is effective for multi-label classification tasks.
- Objectness (OBJ) or Distribution Focal (DFL) Loss: This component helps the model distinguish between foreground objects and the background. It also uses a BCE-based loss to predict the confidence score for each bounding box.

#### 2.4.2. Classification Performance Metrics

The model’s classification performance was evaluated using metrics derived from a confusion matrix, which compares the predicted labels to the ground-truth labels. The four components of this matrix are:

- True Positive (TP): An instance where the model correctly identifies a positive class.
- False Positive (FP): An instance where the model incorrectly identifies a positive class.
- True Negative (TN): An instance where the model correctly identifies a negative class.
- False Negative (FN): An instance where the model incorrectly identifies a negative class.
- From these components, the following metrics were calculated:
- Accuracy: The ratio of all correct predictions (both positive and negative) to the total number of instances, as calculated in Formula (1).

(1) $$\mathrm{Accuracy}=\frac{TP+TN}{TP+FP+TN+FN}$$

- Precision: The ratio of correctly identified positive detections (True Positives, TP) to the total number of positive detections made by the model (TP + False Positives, FP), as shown in Formula (2). It measures the accuracy of the predictions.

(2) $$\mathrm{Precision}=\frac{TP}{TP+FP}$$

- Recall: The ratio of correctly identified positive detections (TP) to the total number of actual positive instances in the data (TP + False Negatives, FN), calculated as shown in Formula (3). It measures the model’s ability to find all relevant objects.

(3) $$\mathrm{Recall}=\frac{TP}{TP+FN}$$

- F1-Score: The harmonic mean of Precision and Recall. It provides a single, balanced measure of a model’s performance, which is particularly useful when there is an uneven class distribution.

(4) $$\mathrm{Recall}=2\;\times \;\frac{Precision\;x\;Recall}{Precision+Recall}$$

#### 2.4.3. Object Detection Metrics

The primary evaluation metric for the object detection task was mean Average Precision (mAP). This metric provides a comprehensive assessment of the model’s ability to both correctly classify and localize objects.

- Average Precision (AP): Calculated as the area under the Precision-Recall curve for a single class, as shown in Formula (5). It summarizes the model’s performance on that specific class across all recall levels

(5) $$\mathrm{AP}=\int_{0}^{1}p\left(r\right)dr$$

- Mean Average Precision (mAP): The mean of the AP values calculated across all classes, as defined by Formula (6). This provides a single, aggregate score for the model’s overall performance. In this study, we report mAP50 (mAP at an IoU threshold of 0.5) and mAP50-95 (mAP averaged over IoU thresholds from 0.5 to 0.95).

(6) $$\mathrm{mAP}=\frac{1}{N}\sum_{i=1}^{N}AP_{i}$$

### 2.5. Implementation Details

All experiments were conducted on a high-performance workstation to ensure computational efficiency and reproducibility. The hardware setup consisted of an Intel Core i9-14900K CPU, 64 GB of DDR5 RAM, and an NVIDIA GeForce RTX 4090 GPU with 24 GB of GDDR6X memory for accelerating the deep learning computations.

The software environment was based on the Windows 11 operating system. The models were implemented in Python (v3.11) using the PyTorch deep learning framework (v2.5.1). We utilized the object detection architectures provided by the Ultralytics library (v8.3.158). The training process was accelerated using NVIDIA’s CUDA Toolkit (v12.1) and cuDNN library (v8.9.2). Data manipulation, image processing, and visualization were handled by standard scientific libraries, including NumPy, OpenCV, and Matplotlib.

## 3. Results

This section presents the empirical results of the study. The evaluation was conducted in two sequential stages, consistent with the two-tiered validation strategy. First, to select the most suitable architecture, the four YOLO model variants were competitively evaluated on the internal validation set. Subsequently, the performance of the selected optimal model was definitively assessed on the separate, independent external test set to determine its generalization capability.

### 3.1. Comparative Performance and Model Selection on the Internal Validation Set

The primary objective of this phase was to identify the optimal model by comparing the performance of YOLOv8x, YOLOv9e, YOLOv10x, and YOLOv11x on the internal validation set. The selection was based on a comprehensive evaluation of detection accuracy, learning efficiency, and computational cost.

The overall object detection performance of the four models was first evaluated using key metrics summarized in Table 4 and Table 5. The YOLOv11x model achieved the highest mean Average Precision at the primary IoU threshold (mAP50 = 0.91), which was the key indicator for overall detection accuracy. Furthermore, it demonstrated a superior balance of precision and recall, culminating in the highest F1-Score of 0.93 among all contestants.

**Figure 4:**
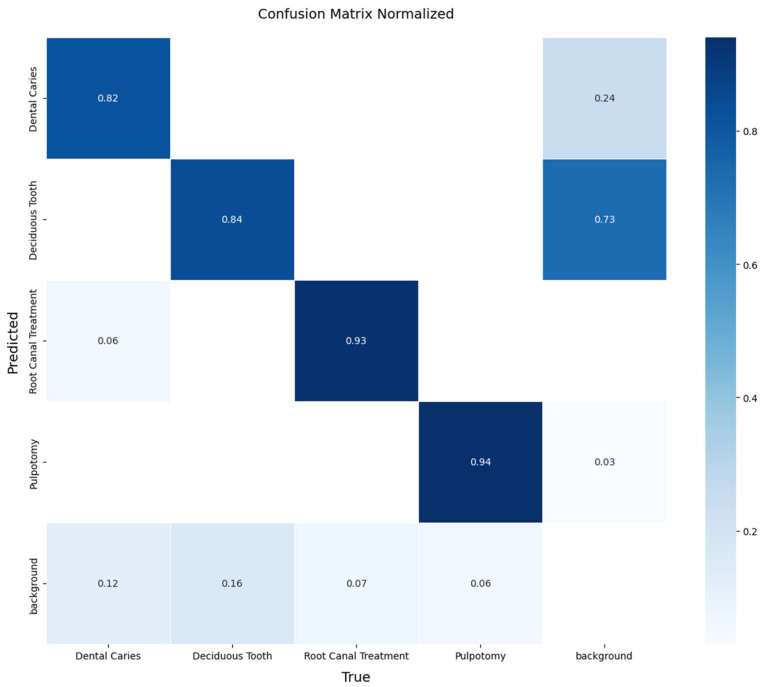
Normalized confusion matrix for the YOLOv11x model on the internal validation set. The matrix illustrates the model’s per-class performance, with the diagonal elements representing the true positive rate (recall) for each class. Off-diagonal elements indicate sources of confusion between classes.

**Figure 5:**
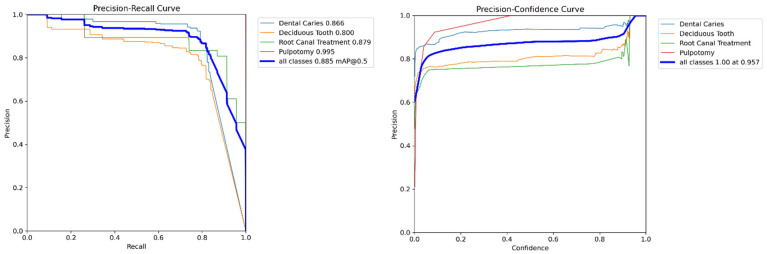
Precision-Recall curve for the YOLOv11x model on the internal validation set. The plot shows the AP for each of the four classes, as well as the overall mAP at an IoU threshold of 0.5.

**Figure 6:**
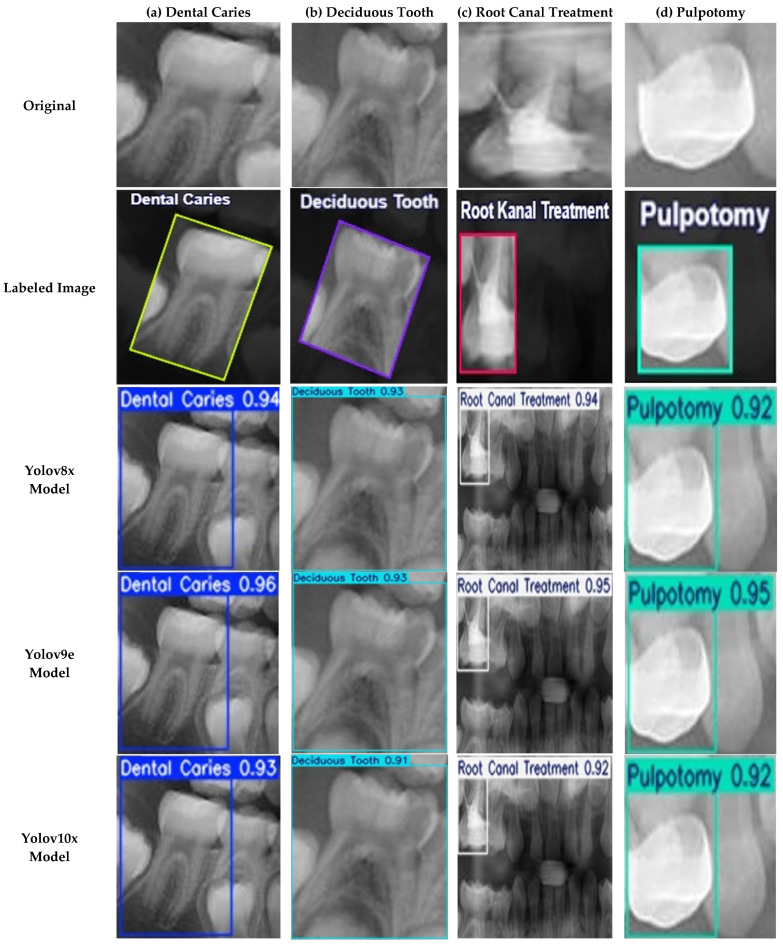

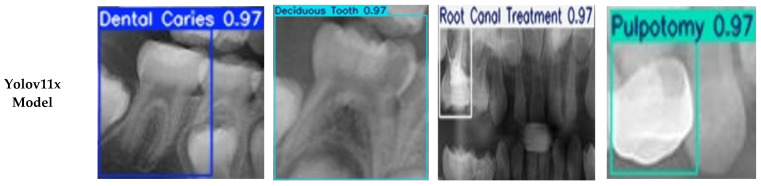
Examples of Successful Detection of YOLO Models for Different Dental Conditions. The images display representative examples of correct detections for (**a**) Dental Caries, (**b**) Deciduous Tooth, (**c**) Root Canal Treatment, and (**d**) Pulpotomy.

A more detailed analysis of the YOLOv11x model’s performance on the validation set provides deeper insights. The model’s per-class accuracy is detailed in the normalized confusion matrix presented in Figure 4. The matrix reveals notable performance in identifying therapeutic interventions, with recall rates of 94% for Pulpotomy and 93% for Root Canal Treatment. For the more visually varied classes, the model achieved robust recall rates of 82% for Deciduous Tooth and 84% for Dental Caries. The relationship between precision and recall is further visualized in Figure 5, where the Precision-Recall curve illustrates the model’s high AP across all classes. Qualitative examples of these successful detections from the validation set are provided in Figure 6.

**Figure 7:**
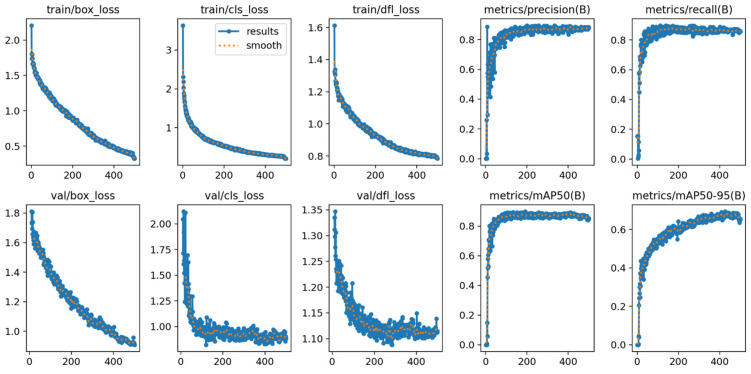
Training and validation learning curves for the YOLOv11x model over 500 epochs. The plots demonstrate the convergence of loss components (box, cls, dfl) and the progression of key performance metrics (mAP, precision, recall), indicating a stable training process without significant overfitting.

Beyond detection accuracy, the models’ learning efficiency was assessed via their validation loss components, as shown in Table 6. Lower loss values signify a more effective learning process. YOLOv11x registered the lowest Box Loss (0.90) and CLS Loss (0.82) values, confirming its superior ability to accurately localize objects and classify them correctly during training. The stability of this training process is demonstrated by the learning curves in Figure 7, which show a consistent decrease in both training and validation loss over 500 epochs, indicating stable convergence without significant overfitting.

Finally, in terms of computational efficiency, all models performed well. As detailed in Table 7, YOLOv11x was among the fastest in terms of training time per epoch (0.90 s).

In summary, based on its leading performance across key detection metrics (mAP50 and F1-Score), its detailed per-class accuracy, its lower validation loss values, and its high computational efficiency, the YOLOv11x model was selected as the optimal model for the final evaluation on the independent external test set.

### 3.2. Final Performance on the Independent External Test Set

After selecting YOLOv11x as the optimal model, its generalization capability was assessed on the independent external test set, which consisted of 150 images the model had never previously encountered. This final evaluation provides a realistic measure of the model’s performance in real-world scenarios.

The overall performance of YOLOv11x on the external test set is summarized in Table 8. The model achieved a robust F1-Score of 0.81, with a recall of 0.83 and a precision of 0.80.

**Figure 8:**
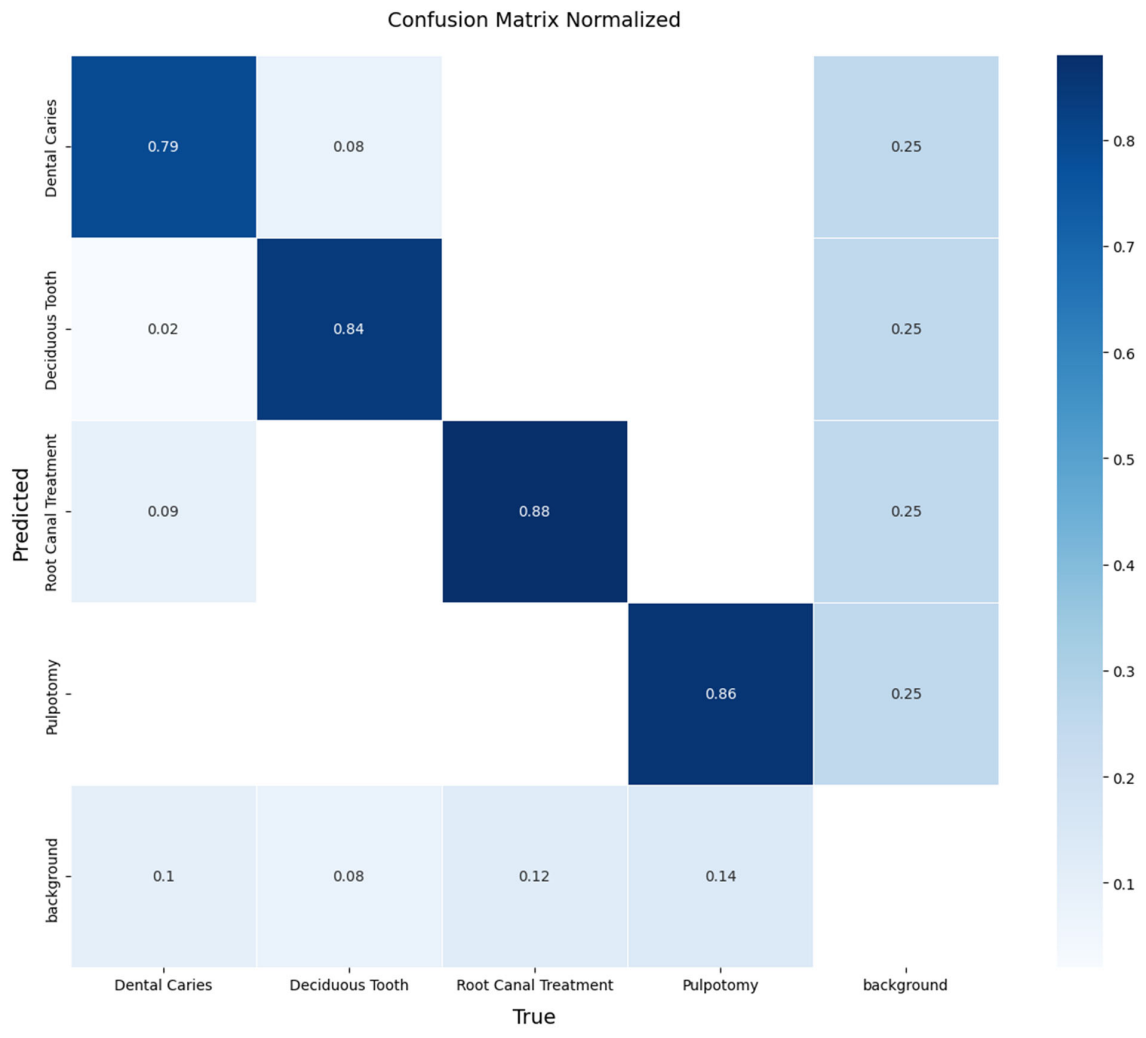
Normalized confusion matrix illustrating the performance of the YOLOv11x model on the independent external test set. The diagonal elements represent the recall (true positive rate) for each class, providing a clear view of per-class accuracy.

A detailed, per-class breakdown of the model’s performance on this unseen data is provided by the normalized confusion matrix in Figure 8. The matrix reveals the model’s performance in detecting therapeutic interventions, achieving a recall of 88% for Root Canal Treatment and 86% for Pulpotomy. The model also demonstrated effective detection for the more challenging classes, achieving a recall of 79% for Dental Caries and 84% for Deciduous Tooth. The off-diagonal values indicate minimal confusion between the primary pathological conditions, with most errors occurring as confusion with the ‘background’ class. These results confirm that the YOLOv11x model can generalize effectively to new data from different sources.

The primary object detection performance on the external test set was evaluated using the mean Average Precision (mAP) metric, with the final scores presented in Table 9. The model achieved a final mAP50 score of 0.82 at the standard IoU threshold of 0.5. For the more stringent evaluation across multiple IoU thresholds (from 0.5 to 0.95), the model maintained a robust mAP50-95 score of 0.62. These mAP scores, which represent a comprehensive assessment of both localization and classification accuracy, provide quantitative evidence of the model’s generalization capability.

## 4. Discussion

This study successfully developed and validated a deep learning model based on the YOLOv11x architecture for the automated detection of four common conditions in pediatric OPG’s. The principal findings demonstrate that the selected model not only performs with high accuracy on internal validation data but also generalizes effectively to a completely independent external test set, highlighting its potential as a reliable clinical decision support tool.

### 4.1. Interpretation of Findings and Clinical Implications

The core achievement of this study is the validated performance of the YOLOv11x model in a challenging, dynamic pediatric imaging environment. Our two-tiered evaluation strategy revealed two key insights. First, the comparative analysis on the internal validation set showed that YOLOv11x offered a validated performance of a leading performance in detection accuracy (mAP50 of 0.91) and learning efficiency compared to other state-of-the-art YOLO variants. Second, and more importantly, the final evaluation on the independent external test set confirmed the model’s clinical potential. The model’s ability to identify therapeutic interventions, with recall rates of 88% for Root Canal Treatment and 86% for Pulpotomy, is a notable finding. This high accuracy is likely attributable to the distinct and consistent radiographic features of these treatments. The model also achieved, clinically relevant recall rates for the more visually ambiguous classes: 79% for Dental Caries and 84% for Deciduous Tooth.

The errors in detecting deciduous teeth, primarily missed detections, are likely concentrated on teeth nearing exfoliation where physiological root resorption diminishes their radiographic signature. This difficulty may be compounded by the superimposition of underlying permanent tooth germs, which can obscure the features of the primary tooth. The few instances of misclassification as dental caries could be attributed to the complex occlusal anatomy of primary molars, which can mimic carious lesions on 2D radiographs.

A detailed analysis of the confusion matrix from the external test (Figure 8) provides deeper insight into specific diagnostic challenges. It shows that most errors were false negatives (confusion with the ‘background’ class) rather than misclassifications between pathological conditions, which suggests the model is conservative in its predictions. Regarding the distinction between primary and permanent teeth, while not a primary objective, the high recall for the ‘Deciduous Tooth’ class and minimal confusion with other classes indicate that the model effectively learned to distinguish primary teeth, though a dedicated study would be needed to confirm its accuracy in numbering or differentiating from all stages of permanent tooth development. As for the minimum degree of caries detected, our annotation protocol focused on radiographically evident lesions that had progressed to the dentin-enamel junction, excluding incipient enamel-only lesions, which defines the current detection threshold of the model.

From a clinical perspective, these findings have several practical implications. An automated system capable of highlighting potential findings with approximately 80–88% recall can serve as a valuable ‘second opinion’ or screening tool for pediatric dentists. In a high-volume clinical setting, this could help reduce diagnostic fatigue and minimize the risk of overlooking common pathologies, particularly on complex mixed dentition radiographs. For instance, the model could flag a tooth with radiographic signs of endodontic treatment, prompting the clinician to cross-reference with the patient’s clinical history. While not a substitute for expert judgment, such a tool has the potential to streamline the diagnostic workflow by drawing the clinician’s attention to specific areas of interest, thereby allowing them to focus more on treatment planning and patient interaction.

### 4.2. Comparison with Existing Literature

The performance of our YOLOv11x model is competitive within the landscape of AI in dental diagnostics. While many studies have focused on adult populations or single pathologies, our work addresses the unique challenges of the pediatric mixed dentition phase. For instance, Kaya et al. [18] achieved an accuracy of 0.89 using YOLOv4 for detecting permanent tooth germs, a task complementary to our detection of deciduous teeth. Our model’s performance is comparable, demonstrating the versatility of the YOLO framework. Similarly, studies using different architectures like Faster R-CNN for primary tooth detection have reported high precision [19], but often lack the inference speed of single-stage detectors like YOLO, which is critical for clinical workflow.

Broader trends in the literature further contextualize our work. A scoping review by Sivari et al. [2] confirms the growing trend and potential of AI in pediatric dentistry, positioning our study as a practical application within this evolving field. Furthermore, a recent study by Bumann et al. [29] focused on segmenting teeth in mixed dentition, achieving a high mAP. Our study complements this by focusing on the detection of specific pathological and therapeutic findings within that same complex environment, demonstrating the feasibility of a multi-target diagnostic tool.

### 4.3. Strengths and Limitations

A primary strength of this study is its rigorous, two-stage validation methodology. By testing our final model on an independent external dataset, we transcended the limitations of a single-source dataset, thereby providing a more realistic and reliable assessment of the model’s generalization capability. Additional strengths include the use of a dual-expert, clinically relevant ground truth, established collaboratively by a radiologist and a board-certified pediatric dentist, and the systematic comparison of four distinct state-of-the-art YOLO architectures

Despite these strengths, we acknowledge certain limitations. Although an external test set was utilized, both datasets were composed of retrospective digital radiographs; consequently, performance may vary on images acquired from different devices or with different exposure parameters. The dataset size, while typical for such studies, could be expanded to further improve model robustness. An additional limitation is the inherent class imbalance within the dataset (e.g., a higher prevalence of ‘Dental Caries’ labels compared to ‘Pulpotomy’), which can potentially hinder the learning process for minority classes. Moreover, in its current iteration, our model is designed for detection identifying the presence of findings rather than for classification of severity or grading of conditions, such as caries depth or treatment quality.

Finally, the study’s scope was intentionally focused on comparing architectures within the state-of-the-art YOLO family to identify the optimal single-stage detector for this task. Consequently, a direct comparative analysis against other types of architectures, such as segmentation models (e.g., U-Net) or two-stage detectors, was not performed. While our results validate the effectiveness of the YOLO framework, a broader comparative study could yield further insights and represents a promising direction for future research.

### 4.4. Future Directions

The promising outcomes of this study lay the foundation for several future research avenues. To further enhance the model’s generalization capability, future work should focus on training with larger and more diverse datasets sourced from multiple clinical centers. Exploring advanced data augmentation techniques and architectural modifications, such as the integration of more sophisticated attention mechanisms, could notably improve the detection of ambiguous carious lesions and mitigate confounding from background structures.

A critical next step involves conducting prospective clinical trials to assess the model’s impact on real-world diagnostic accuracy, treatment planning, and clinical workflow efficiency for pediatric dentists. Furthermore, a significant advancement would be to evolve the model from its current “detection” capability into a tool for “severity assessment,” capable of classifying carious lesions (e.g., Enamel-Dentin-Pulp involvement) or determining the grades of root resorption. Finally, investigating the model’s utility for longitudinal tracking of a patient’s dynamic dental development and optimizing its performance for deployment on clinical computers with varying hardware capacities will be invaluable steps toward its seamless clinical integration.

## 5. Conclusions

This study successfully developed and, most importantly, rigorously validated a deep learning framework using the YOLOv11x architecture for the automated detection of four critical findings in pediatric OPG’s. The selected model demonstrated not only high accuracy on internal validation data but also robust generalization capabilities when evaluated on a completely independent external test set. The model’s ability to reliably detect diverse conditions, from carious lesions to therapeutic interventions, underscores its potential as a powerful diagnostic aid for clinicians. By leveraging a state-of-the-art, single-stage detector and demonstrating its performance through a stringent two-tiered validation process, this work highlights the potential of AI-driven systems to assist clinicians in the demanding environment of pediatric dentistry. The developed model can serve as a valuable supplementary tool, aiming to improve the consistency of detection and support workflow efficiency, thereby taking a step toward the broader goal of enhancing dental healthcare outcomes for children.

## Figures and Tables

**Table 1:** Distribution and relative frequency of the four selected dental conditions within the primary training and validation dataset derived from Zhang et al. [20] (*n* = 644 images).

	Frequency (Label)	Relative Frequency (%)	Cumulative (%)
Dental Caries	830	43.57	43.57
Deciduous Tooth	791	41.52	85.09
Root Canal Treatment	171	8.98	94.07
Pulpotomy	113	5.93	100.00

**Table 2:** Comparison of computational costs and model parameters for the evaluated YOLO variants (YOLOv8x, YOLOv9e, YOLOv10x, and YOLOv11x).

Algorithms	Pixels	Parameters (M)	FLOPs (G)
YOLOv8x	640	68.16	311.2
YOLOv9e	640	58.1	192.5
YOLOv10x	640	29.5	160.4
YOLOv11x	640	56.9	194.9

**Table 3:** Detailed hyperparameters and configuration settings for model training.

Category	Parameter	Value/Description
Dataset & Input	Input Image Size	640 × 640 pixels
	Batch Size	16
Training Regimen	Number of Epoch	500
Optimizer & Learning Rate	Optimizer	SGD (Stochastic Gradient Descent)
	Momentum	0.937
	Weight Decay	0.0005
	Initial Learning Rate (lr0)	0.01
	Learning Rate Scheduler	Cosine Annealing
Data Augmentation	Mosaic	Applied to combine four training images into one.
	MixUp	Applied to create composite images by linearly interpolating two images and their labels.
	Random Affine Transformations	Included random rotations, scaling, and translations.
Loss & Evaluation	Training IoU Threshold	0.5
	Loss Function Components	A composite function including: Box Loss: Complete IoU (CIoU) Loss Class Loss: Binary Cross-Entropy (BCE) Objectness Loss: Distribution Focal Loss (DFL)

**Table 4:** Performance comparison of the evaluated YOLO model variants on the internal validation set.

	Accuracy	Recall	Precision	F1-Score
YOLOv8x	0.86	0.89	0.92	0.90
YOLOv9e	0.87	0.89	0.91	0.90
YOLOv10x	0.87	0.89	0.92	0.90
YOLOv11x	0.91	0.92	0.94	0.93

**Table 5:** mAP scores for the evaluated YOLO model variants on the internal validation set. The mAP50 metric was the primary indicator for model selection.

	mAP50	mAP50-95
YOLOv8x	0.89	0.71
YOLOv9e	0.90	0.70
YOLOv10x	0.89	0.71
YOLOv11x	0.91	0.69

**Table 6:** Comparison of validation loss components (Box, CLS, and DFL) for each model on the internal validation set.

	Box Loss	CLS Loss	DFL Lose
YOLOv8x	0.93	0.83	1.27
YOLOv9e	0.92	0.84	1.49
YOLOv10x	1.84	2.38	2.23
YOLOv11x	0.90	0.82	1.10

**Table 7:** Training and validation time per epoch for each YOLO model variant.

	Training Time Per Epoch (s)	Validation Time per Epoch (s)
YOLOv8x	0.92	0.89
YOLOv9e	0.91	0.89
YOLOv10x	0.92	0.89
YOLOv11x	0.90	0.90

**Table 8:** Final performance of the selected YOLOv11x model on the independent external test set (*n* = 150 images). The table reports the primary object detection metric (mAP) and key classification metrics.

	Accuracy	Recall	Precision	F1-Score
YOLOv11x	0.82	0.83	0.80	0.81

**Table 9:** mAP scores for YOLOv11x model variants assessed in the test set.

	mAP50	mAP50-95
YOLOv11x	0.82	0.62

## Data Availability

The primary public dataset from Zhang et al. [20], used for model training and internal validation, is accessible at: https://www.kisa.link/PAhjU (accessed on 20 November 2024). The external test dataset was curated from the following publicly available projects on the Roboflow Universe platform. The specific links to the source projects are: https://www.kisa.link/wMjfV (accessed on 10 April 2025); https://www.kisa.link/TsHld (accessed on 10 April 2025); https://www.kisa.link/RkaVO (accessed on 12 April 2025).
